# An orally-active adiponectin receptor agonist mitigates cutaneous fibrosis, inflammation and microvascular pathology in a murine model of systemic sclerosis

**DOI:** 10.1038/s41598-018-29901-w

**Published:** 2018-08-07

**Authors:** Takashi Yamashita, Katja Lakota, Takashi Taniguchi, Ayumi Yoshizaki, Shinichi Sato, Wen Hong, Xingchun Zhou, Snezn Sodin-Semrl, Feng Fang, Yoshihide Asano, John Varga

**Affiliations:** 1 Department of Dermatology, University of Tokyo Graduate School of Medicine, Tokyo, Japan; 2 Department of Rheumatology, University Medical Centre Ljubljana, Ljubljana, Slovenia; 3 Northwestern Scleroderma Program, Feinberg School of Medicine, Chicago, IL USA

## Abstract

The hallmarks of systemic sclerosis (SSc) are autoimmunity, microangiopathy and fibrosis. Skin fibrosis is accompanied by attrition of the dermal white adipose tissue layer, and alterations in the levels and function of adiponectin. Since these findings potentially implicate adiponectin in the pathogenesis of SSc, we employed a novel pharmacological approach to augment adiponectin signaling using AdipoRon, an orally active adiponectin receptor agonist. Chronic treatment with AdipoRon significantly ameliorated bleomycin-induced dermal fibrosis in mice. AdipoRon attenuated fibroblast activation, adipocyte-to-myofibroblast transdifferentiation, Th2/Th17-skewed polarization of the immune response, vascular injury and endothelial-to-mesenchymal transition within the lesional skin. *In vitro*, AdipoRon abrogated profibrotic responses elicited by TGF-β in normal fibroblasts, and reversed the inherently-activated profibrotic phenotype of SSc fibroblasts. In view of these broadly beneficial effects on all three cardinal pathomechanisms underlying the clinical manifestations of SSc, pharmacological augmentation of adiponectin signaling might represent a novel strategy for the treatment of SSc.

## Introduction

Systemic sclerosis (SSc) is a chronic multi-factorial disease characterized by early inflammation, followed by microangiopathy and fibrosis in the skin and internal organs^1^. While fibroblast activation elicited by transforming growth factor (TGF)-β, chemokines and other extracellular cues is recognized as an essential early step in the development of fibrosis, the factors driving persistent vasculopathy, loss of intradermal adipose tissue and chronic unresolving fibrosis in SSc are not well understood^2,3^. White adipose depots, widely distributed throughout the body, are comprised of hormonally active adipocytes producing a range of adipokines with pleiotropic functions. Secreted adipokines exert potent paracrine and humoral effects on a variety of cells, and regulate inflammatory cell accumulation and endothelial activation in diverse pathological processes^4–7^. Recent studies have generated substantial evidence implicating deregulated adipocyte function and adipokine secretion as pathogenic factors in fibrosis and SSc.

A pathologic hallmark of SSc is accumulation in the lesional tissue of activated myofibroblasts that are responsible for excessive extracellular matrix (ECM) deposition and tissue remodeling. Profibrotic myofibroblasts might originate from resident fibroblasts, or via *in situ* transdifferentiation of other cell lineages resident within the fibrotic microenvironment^8^. Recent studies identified adipocytic cells within intradermal adipose tissue as another potential source of activated myofibroblasts during tissue injury^6,9^. Since differentiated adipocytes produce adipokines that help maintain tissue homeostasis through autocrine, paracrine and endocrine mechanisms^10^, altered adipokine balance resulting from adipocyte loss or dysfunction might contribute to inflammation, vascular dysregulation and fibrosis characteristic of SSc^4,11–20^. Adiponectin is a 244-amino acid adipokine that is synthesized almost exclusively by differentiated white adipocytes^21,22^. In distinction from other adipokines, adiponectin is paradoxically reduced in obesity. The consequent chronic hypoadiponectinemia and adiponectin dysfunction contribute to the pathogenesis of type 2 diabetes, metabolic syndrome, cardiovascular and liver disease and psoriasis. Levels of adiponectin have been shown to be elevated in autoimmune and inflammatory diseases including systemic lupus erythematosus^23^, rheumatoid arthritis^24^, inflammatory bowel disease^25^, and type 1 diabetes^25^. In contrast, circulating adiponectin levels are decreased in SSc, particularly in patients with early-stage disease^4,11,12,20^. Moreover, adiponectin levels inversely correlate with the skin score, a marker for SSc skin involvement. Taken together with its potent pleiotropic biological activities, including anti-inflammatory and anti-fibrotic effects^26–29^, deregulated adiponectin signaling is likely to have a role in SSc pathogenesis.

Adiponectin and synthetic adiponectin mimetics have been shown to exert protective effects in preclinical models of pulmonary, cardiac, and hepatic fibrosis^27–29^. AdipoRon is a novel orally-active small molecule that serves as a potent selective agonist of the AdipoR1 and AdipoR2 adiponectin receptors^30^. Recent studies showed that AdipoRon had beneficial effects on myocardial ischemia/reperfusion injury^31^, and promoted vasorelaxation^32^. The present studies sought to investigate the effects of AdipoRon in an experimental model of SSc that recapitulates inflammatory, vascular and fibrotic features of the human disease. The results indicate that AdipoRon treatment attenuated inflammation, microvascular injury and fibrosis in the skin, and abrogated TGF-β-mediated and constitutive activation of normal and SSc fibroblasts. The beneficial effects of AdipoRon on all three cardinal pathogenic pathways implicated in SSc suggest that pharmacological activation of adiponectin receptors might represent an innovative therapeutic approach to SSc.

## Results

### AdipoRon prevents dermal fibrosis induced by bleomycin

**Figure 1:**
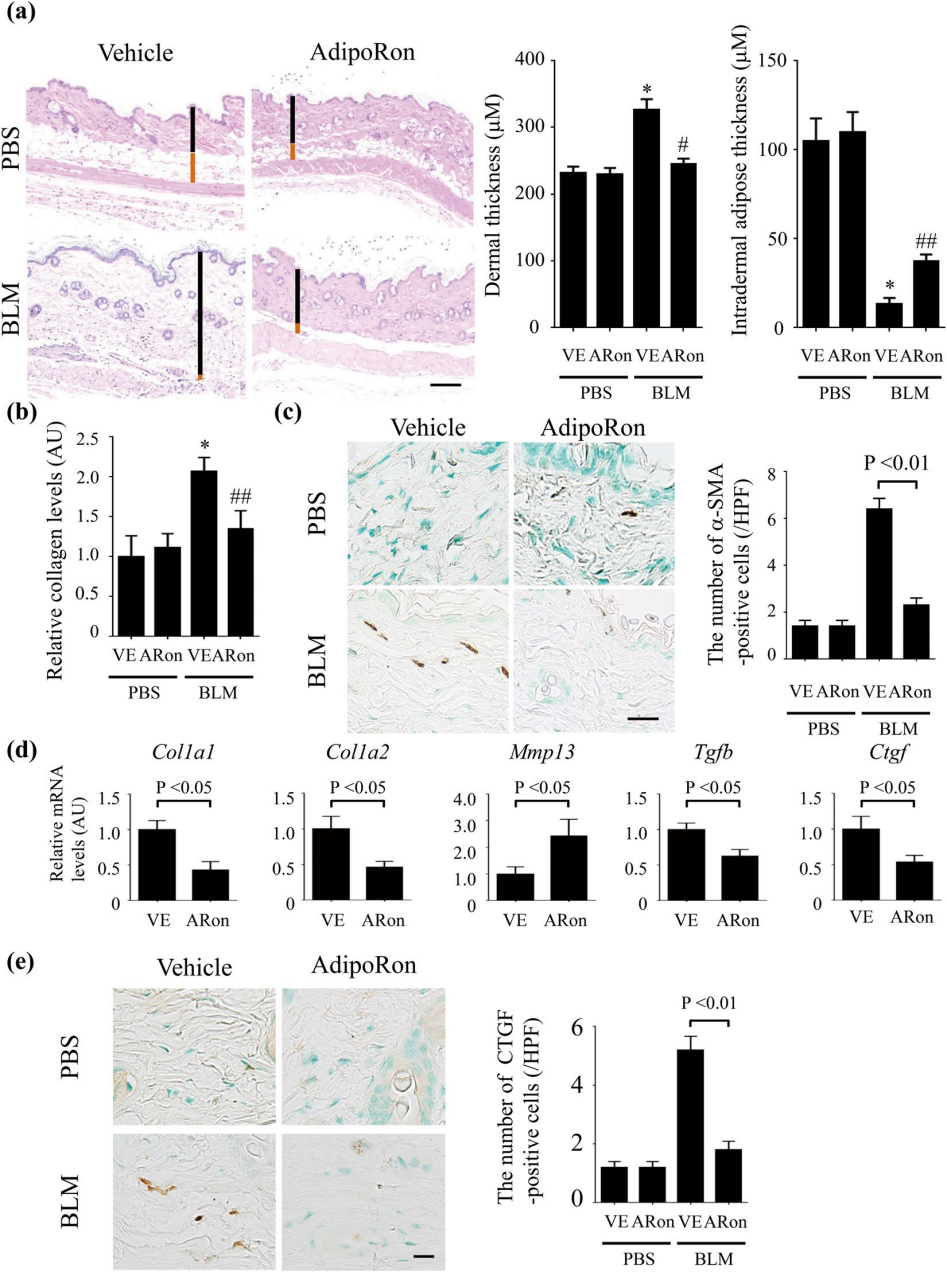
AdipoRon prevents the development of dermal fibrosis. (**a**) Skin sections of bleomycin (BLM)- or PBS-injected mice treated with AdipoRon or vehicle were evaluated by hematoxylin & eosin staining (n = 4–10 mice/group). Representative images. Vertical black and red bars indicate thickness of dermis and subcutaneous adipose tissue, respectively. (**b**) Collagen contents were measured by hydroxyproline assay (n = 4–10 mice/group). (**c**,**e**) The number of α-smooth muscle actin (α-SMA)-positive (**c**) and CTGF-positive cells (**e**) was determined by immunohistochemistry (n = 10 mice/group). The numbers of positive cells per high power field are shown. (**d**) mRNA levels of fibrosis-related genes were determined by qRT-PCR in the lesional skin (n = 6–9 mice/group). The graphs represent mean ± SEM of indicated parameters. Scale bars are 100 μm for A and 20 μm for C and E. *p < 0.01 compared with mice treated with PBS and vehicle. ^#^p < 0.01 compared with mice treated with BLM and vehicle. ^##^p < 0.05 compared with mice treated with BLM and vehicle. AU, arbitrary unit; HPF, high power field; ARon, AdipoRon; VE, vehicle.

To investigate the effect of AdipoRon in a murine model of SSc, mice were randomly assigned to treatment with vehicle, bleomycin, or those in combination with AdipoRon. Prolonged administration of AdipoRon (50 mg/kg) for up to 28 days was well tolerated and no signs of toxicity were observed. AdipoRon treatment significantly attenuated the increase in dermal thickness induced by bleomycin, while augmenting the dermal white adipose tissue layer (Fig. 1a). Collagen content and the number of myofibroblasts within the lesional skin were significantly reduced in mice treated with AdipoRon (Fig. 1b,c). Additionally, transdifferentiation of intradermal adipocytes to myofibroblasts, detected by double immunofluorescence for α-SMA and perilipin, was reduced (Supplementary Fig. 1). Levels of *Col1a1* and *Col1a2* mRNA in the skin were reduced in AdipoRon-treated mice, while *Mmp13* mRNA levels were significantly elevated (Fig. 1d). Since TGF-β1 and connective tissue growth factor (CTGF) are recognized as critical mediators of fibrosis^33^, we examined the expression of these growth factors in the lesional skin. The results showed a significant decrease in *Tgfb1* and *Ctgf* gene expression in mice treated with AdipoRon (Fig. 1d), which was also confirmed by immunohistochemistry (Fig. 1e and Supplementary Table 1). Taken together, these findings indicate that chronic AdipoRon treatment exerts a potent anti-fibrotic effect in mice by attenuating myofibroblast transition, adipocyte-to-myofibroblast transdifferentiation, collagen accumulation and expression of key pro-fibrotic growth factors within the lesional skin. Importantly, bleomycin increased the expression of adiponectin receptors, AdipoR1 and AdipoR2, in dermal fibroblasts, endothelial cells, inflammatory cells, and adipocytes irrespective of AdipoRon treatment (Supplementary Fig. 2 and Supplementary Table 2), and AdipoRon treatment increased the phosphorylation of adenosine monophosphate-activated protein kinase (AMPK), a major intracellular mediator of adiponectin signaling, in skeletal muscles from both control (PBS-treated) and bleomycin-treated mice (Supplementary Fig. 3), suggesting that AdipoRon at least in part directly exerts its effect on bleomycin-induced skin fibrosis.

### AdipoRon attenuates fibroblast activation

**Figure 2:**
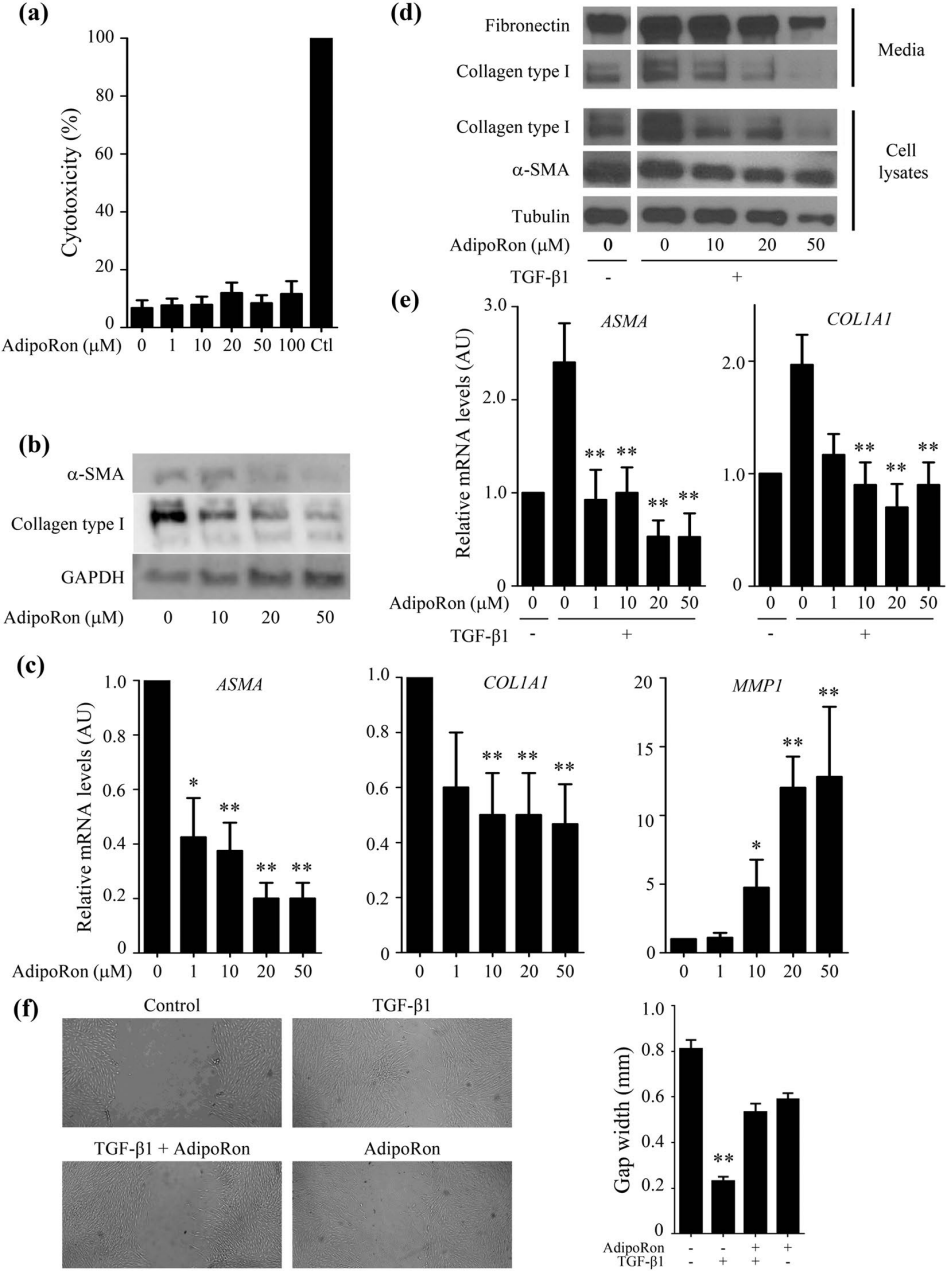
AdipoRon inhibits dermal fibroblast activation. Foreskin fibroblasts at confluence were incubated in serum-free media with indicated concentrations of AdipoRon for 24 h. In indicated experiments cells were incubated in the presence of TGF-β1 (5 ng/ml). (**a**) Cytotoxicity was evaluated by LDH assays. (**b**) Whole cell lysates were subjected to immunoblotting. (**c**,**e**) Total RNA was subjected to qRT-PCR. (**d**) Whole cell lysates and culture media were subjected to immunoblotting. Full-length blots are presented in Supplementary Fig. 4. (**f**) *In vitro* wound healing assays. Cultures were incubated with TGF-β1 and 20 μM of AdipoRon. Gap widths were determined at 24 h using ImageJ. For A, C, and E, bars represent mean ± SEM of triplicate determinations. For B and D, images representative from three independent experiments were shown. For F, bars represent mean ± SEM of six determinations from three randomly selected fields. *p < 0.05 versus cells without any treatment. **p < 0.01 versus cells without any treatment for C and F, and cells treated with TGF-β1 alone for E. Ctl, control; AU, arbitrary unit.

The molecular mechanism underlying the anti-fibrotic properties of AdipoRon were further investigated *in vitro* with normal human dermal fibroblasts. Initial studies established the absence of cytotoxicity of AdipoRon at concentrations up to 50 μM (Fig. 2a); therefore, all subsequent experiments were performed with AdipoRon at concentrations ≤50 μM. Incubation of confluent fibroblasts with AdipoRon for 24 h resulted in dose-dependent down-regulation of type I collagen, fibronectin and α-SMA, while expression of MMP-1 was increased, as expected^34^ (Fig. 2b–d, full-length blots of Fig. 2d in Supplementary Fig. 4). Significantly, AdipoRon abrogated TGF-β1-induced stimulation of type I collagen and α-SMA gene expression in a dose-dependent manner (Fig. 2e). Furthermore, *in vitro* wound-healing assay demonstrated that treatment with AdipoRon abrogated the increase in fibroblast migration elicited by TGF-β1 (Fig. 2f).

**Figure 3:**
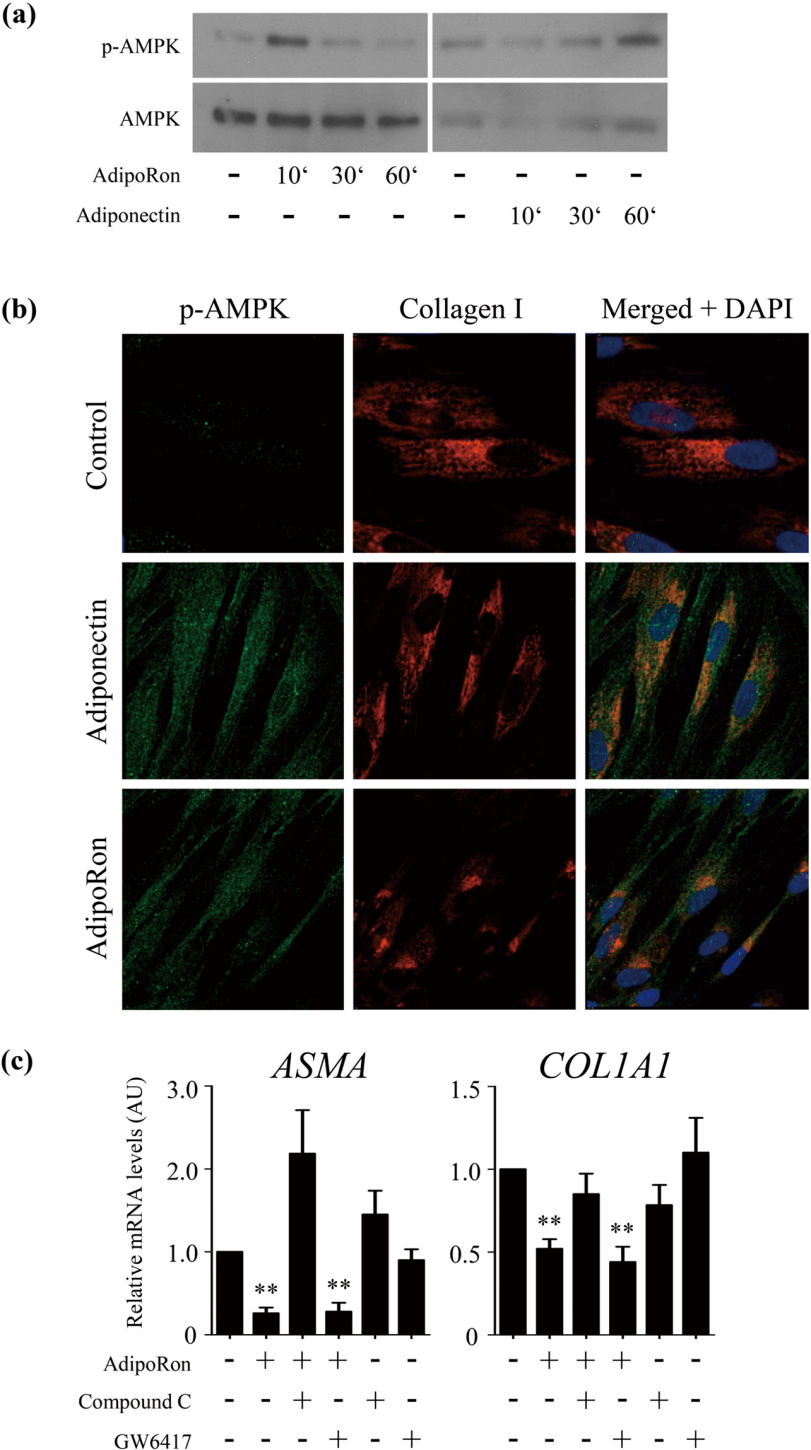
AdipoRon blocks TGF-β-dependent fibrotic responses. Foreskin fibroblasts at confluence were incubated in serum-free media with AdipoRon (20 μM) or adiponectin (10 ng/ml) for indicated periods. (**a**) Whole cell lysates were subjected to immunoblotting. (**b**) Fibroblasts incubated with adiponectin or AdipoRon were immunostained with antibodies to collagen Type I (red), p-AMPK (green) and DAPI (blue). Representative images from three independent experiments are shown. (**c**) Cultures were preincubated with Compound C (5 μM) or GW6417 (1 μM) for 1 h prior to 24-h AdipoRon treatment, and mRNA levels were determined by qRT-PCR. The graphs represent mean ± SEM from at least three experiments. **p < 0.01 versus untreated control cells.

To further explore the anti-fibrotic mechanism of AdipoRon, we next focused on downstream signaling pathways implicated in adiponectin receptor signaling. AMPK and peroxisome proliferator-activated receptor-α (PPARα) are cardinal mediators for adiponectin signal transduction in fibroblasts^5,35^. AdipoRon treatment of normal fibroblasts resulted in a rapid increase in AMPK phosphorylation, and was relatively short-lived (Fig. 3a). Intriguingly, the AdipoRon effect on AMPK was more rapid than that elicited by adiponectin, possibly due to slight differences in strength/affinity ligand binding to receptors. Immunocytochemistry further demonstrated that in normal fibroblasts, AdipoRon treatment caused a reduction in cellular type I collagen, while increasing p-AMPK; fibroblasts showing the highest levels of p-AMPK exhibited signficant reductions in collagen levels (Fig. 3b). The anti-fibrotic effects of AdipoRon were abrogated by pretreatment of the cultures with Compound C, a selective AMPK inhibitor, while GW6417, a selective inhbitor of PPARα, showed no effect (Fig. 3c). These observations support the importance of AMPK activation as a possible mechanism to account for the anti-fibrotic effect of AdipoRon.

### AdipoRon inhibits Th2/Th17-skewed immune polarization induced by bleomycin

**Figure 4:**
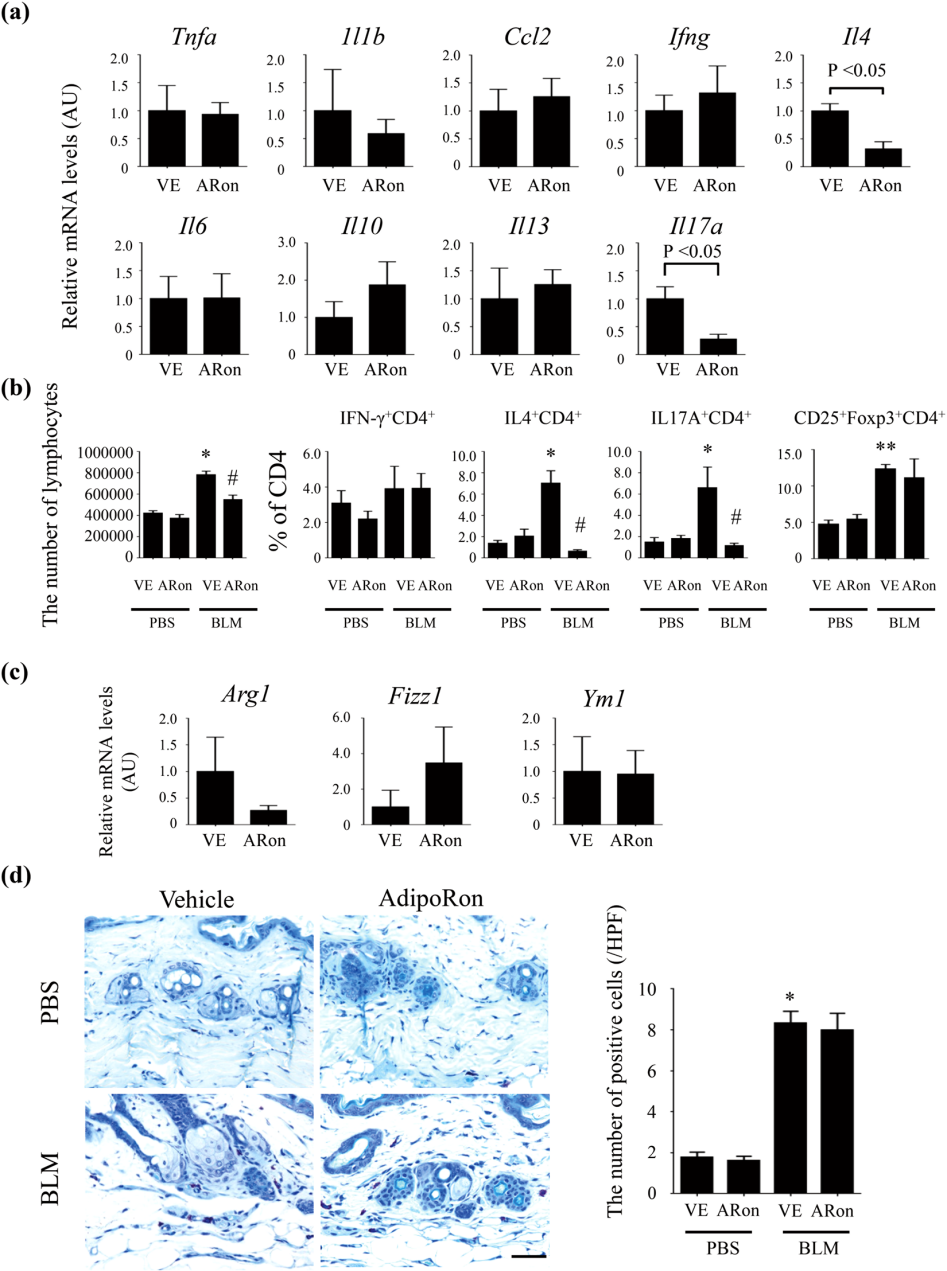
AdipoRon inhibits Th2/Th17-skewed immune polarization in bleomycin-treated mice. (**a**–**d**) Mice were injected with bleomycin (BLM) or PBS for one week alone or together with AdipoRon. *Tnfa*, *Il1b*, *Ccl2*, *Ifng*, *Il4*, *Il6*, *Il10*, *Il13*, and *Il17a* mRNA levels in the lesional skin were measured by qRT-PCR (n = 4–6 mice/group, **a**). The total number of lymphocytes and the proportions of IFN-γ-, IL-4-, and IL-17A-producing cells and Foxp3^+^ cells in CD4^+^ T cells were determined by flow cytometry with cells from skin-draining lymph nodes (n = 5–6 mice/group, **b**). mRNA expression of the *Arg1*, *Fizz1*, and *Ym1* genes was evaluated in the lesional skin by qRT-PCR (n = 4–6 mice/group, **c**). The number of toluidine blue-positive cells was determined by immunostaining (n = 4–6 mice/group, **d**). Each graph represents mean ± SEM of the indicated parameter. Representative images are shown. A scale bar is 20 μm. *p < 0.01 compared with mice treated with PBS and vehicle. **p < 0.05 compared with mice treated with PBS and vehicle. ^#^p < 0.01 compared with mice treated with BLM and vehicle. AU, arbitrary unit; HPF, high power field; ARon, AdipoRon; VE, vehicle.

Since fibroblast activation by inflammatory cells and their secreted product plays an important pathogenic role in bleomycin-induced skin fibrosis^8^, we next focused on the effect of AdipoRon on inflammatory responses. To this end, we evaluated *in vivo* changes in cutaneous cytokines and chemokines at an early time point (7 days) when skin fibrosis is still relatively modest. A significant reduction in *Il4* and *Il17a* mRNA levels was seen in AdipoRon-treated mice, indicating suppression of Th2 and Th17-dominant immune responses, while levels of the *Ifng* and *Il10* mRNA were modestly elevated (Fig. 4a). Flow cytometry of cells isolated from skin-draining lymph nodes showed a decrease in the total numbers of lymphocytes and decreased proportions of IL-4- and IL-17A-producing CD4^+^ T cells in mice treated with AdipoRon, while the proportions of IFN-γ-producing and Foxp3^+^ cells were unaltered (Fig. 4b). In view of the critical role of M2 macrophages and mast cells in SSc fibrosis^36–38^, we assessed the impact of AdipoRon on these cell populations. The results showed that expression of the M2 macrophage markers, such as *Ang1*, *Fizz1* and *Ym1*, and the numbers of toluidine blue-positive cells in the lesional skin were unchanged upon treatment with AdipoRon (Fig. 4c,d).

### AdipoRon treatment mitigates ICAM-1 expression in microvascular endothelial cells

**Figure 5:**
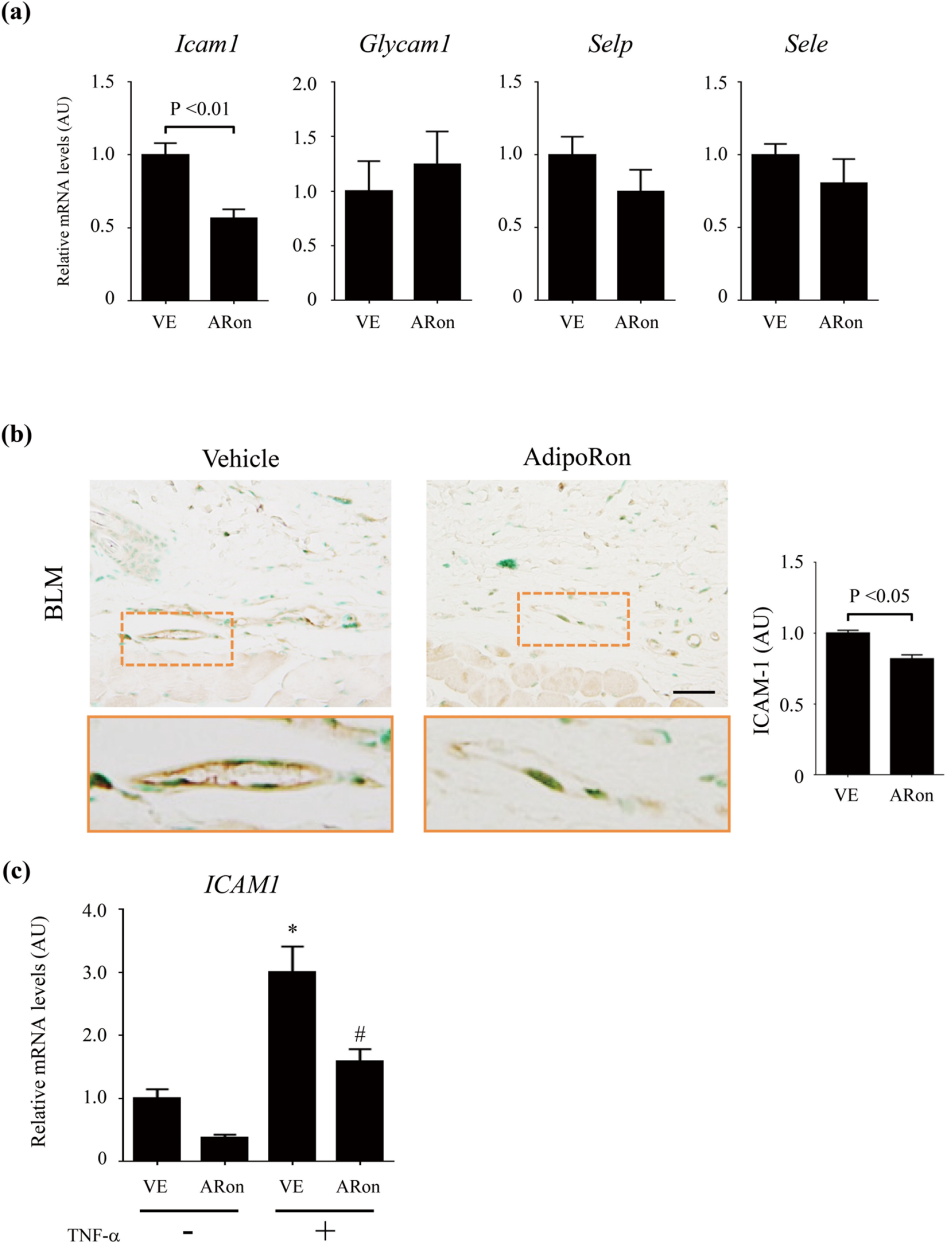
AdipoRon attenuates ICAM-1 in dermal microvascular endothelial cells. Mice were treated with bleomycin (BLM) or PBS for 4 weeks alone or combined with AdipoRon. (**a**,**c**) mRNA expression of cell adhesion molecules in lesional skin (**a**) and *ICAM1* mRNA expression in human dermal microvascular endothelial cells (HDMECs) treated *ex vivo* with TNF-α (5 ng/ml) in the presence or absence of AdipoRon (20 μM) (**c**) were assessed by qRT-PCR (n = 6–9). (**b**) Lesional skin sections were immunostained with anti-ICAM-1 antibody (n = 4–6 mice/group). Representative images. Lower panels correspond to areas indicated by dotted lines. Signal intensity was analyzed by ImageJ. Each bar represents the mean ± SEM of indicated parameter. *p < 0.01 versus control-PBS group. ^#^p < 0.01 versus *Fli1* ECKO-PBS group. AU, arbitrary unit. ARon, AdipoRon; VE, vehicle.

We had previously demonstrated that Th2/Th17 cell infiltration in the dermis is governed by the balance between intercellular adhesion molecule 1 (ICAM-1) and glycosylation-dependent cell adhesion molecule 1 (GlyCAM-1) acting as positive regulators, and P-selectin and E-selectin acting as negative regulators^39^. We therefore next investigated the impact of AdipoRon on the expression of these cell adhesion molecules. Initial experiments confirmed expression of AdipoR1 and AdipoR2 in endothelial cells (Supplementary Fig. 5). The increase in *Icam1* mRNA levels in bleomycin-treated mice was mitigated by AdipoRon, while those of the *Glycam1*, *Selp*, and *Sele* genes were not altered (Fig. 5a). Immunohistochemistry confirmed that in bleomycin-treated mice AdipoRon markedly attenuated ICAM-1 levels in small vessels of the lesional skin (Fig. 5b). Consistent with these *in vivo* observations, we found that treatment of human dermal microvascular endothelial cells (HDMECs) with AdipoRon *ex vivo* abrogated the stimulation of *ICAM1* mRNA induced by TNF-α (Fig. 5c). These results suggest that the inhibitory effects of AdipoRon on Th2/Th17 cell accumulation within the lesional skin in bleomycin-treated mice could be mediated, at least in part, by preventing ICAM-1 expression of endothelial cells.

### AdipoRon treatment attenuates endothelial-to-mesenchymal transition in the lesional skin of bleomycin-treated mice

**Figure 6:**
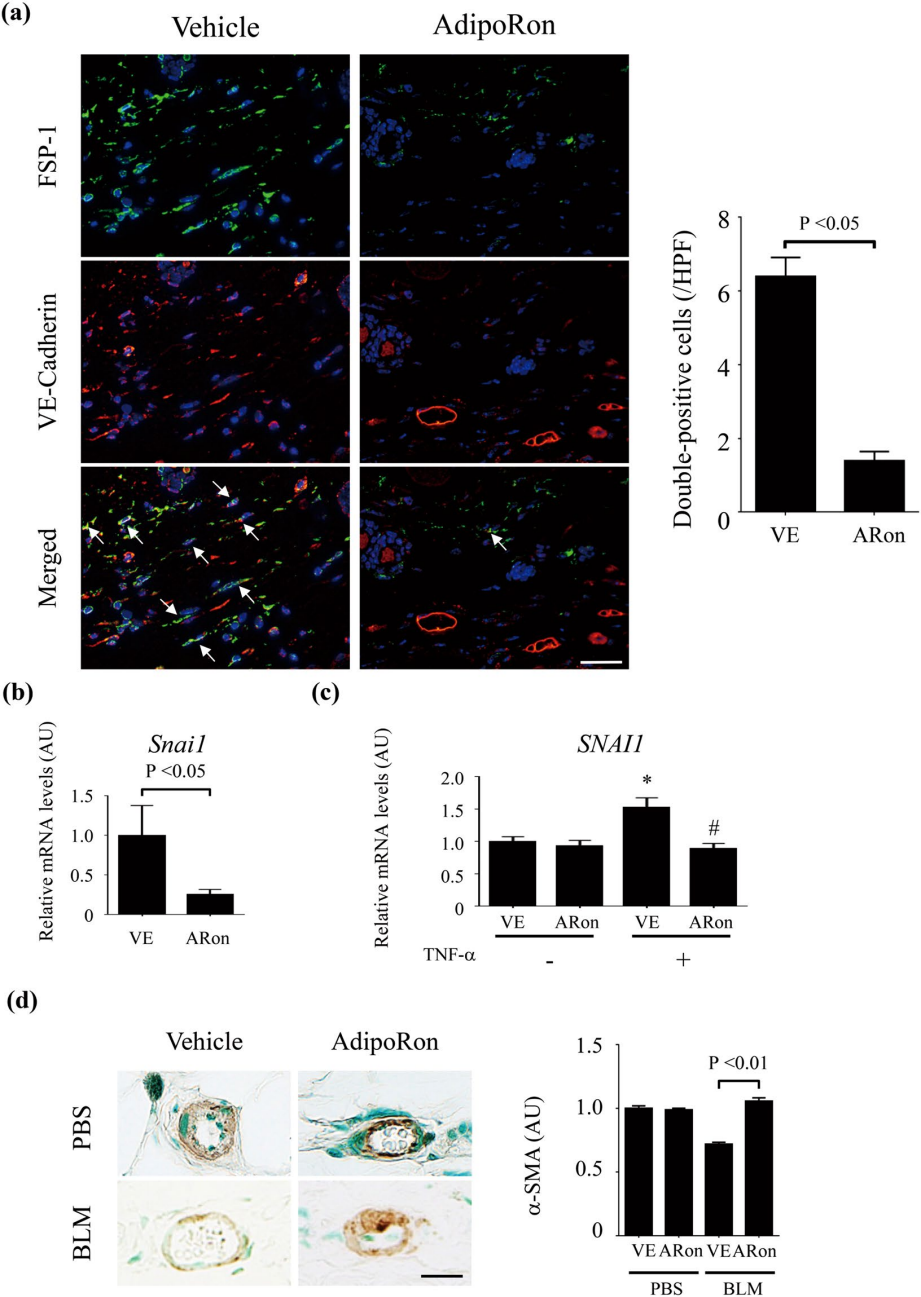
AdipoRon treatment attenuates endothelial-to-mesenchymal transition in the lesional skin. Mice were treated with bleomycin (BLM) or PBS for 4 weeks alone or combined with AdipoRon. (**a**) Immunofluorescence staining of FSP-1 (green) and VE-cadherin (red); FSP-1/VE-cadherin double-positive cells indicated by arrows. Representative images. Right panel, the number of double-positive cells was determined (n = 5 mice/group). (**b**,**c**) *Snail1* mRNA expression determined by qRT-PCR in the lesional skin (n = 8–9 mice/group, **b**); and in HDMECs treated *ex vivo* with TNF-α (5 ng/ml) in the presence or absence of AdipoRon (20 μM) (n = 6, **c**). (**d**) Immunostaining for α-smooth muscle actin (α-SMA) in lesional skin from BLM- or PBS-treated mice (n = 4–10 mice/group). α-SMA-positive cells in vascular walls are recognized as pericytes. Signal intensity of α-SMA determined by ImageJ. Scale bars are 20 μm. Bars represent mean ± SEM of indicated parameters. *p < 0.01 compared with cells treated with vehicle alone. ^#^p < 0.01 compared with cells treated with vehicle and TNF-α. AU, arbitrary unit; HPF, high power field; ARon, AdipoRon; VE, vehicle.

Vascular injury in the bleomycin model of SSc is thought to contribute to the accumulation of interstitial myofibroblasts through the process of EndoMT^40^. Therefore, we evaluated the impact of AdipoRon treatment on this cellular transition by double immunofluorescence for VE-cadherin and fibroblast-specific protein 1 (FSP1), a validated indicator of EndoMT *in vivo*^41–44^. As previously reported, increased number of spindle-shaped double-positive cells was readily observed in the lesional skin from bleomycin-treated mice^15,40,45,46^. AdipoRon treatment markedly attenuated the accumulation of double-positive cells (Fig. 6a). Furthermore, mRNA expression of *Snail1*, encoding a master regulator of EndoMT, was significantly attenuated (Fig. 6b). Parallel *in vitro* experiments with HDMECs showed that AdipoRon effectively suppressed enhanced *SNAIL1* mRNA expression induced by TNF-α (Fig. 6c). Since EndoMT is closely associated with vascular destabilization^46^, we next evaluated stability of dermal microvessels by assessing phenotypical alteration of pericytes using immunostaining for α-SMA, a marker of pericytes with angiostatic phenotype, and platelet-derived growth factor receptor β (PDGFRβ), a marker of pericytes with pro-angiogenic phenotype. As previously reported, α-SMA expression was decreased^15,45^, while PDGFRβ expression was increased in dermal vessels of the lesional skin, indicating that bleomycin promotes a pro-angiogenic state (left panels of Fig. 6d and Supplementary Fig. 6). Of note, AdipoRon treatment reversed the expression profile of α-SMA and PDGFRβ in dermal small vessels of bleomycin-treated mice (right panels of Fig. 6d and supplementary Fig. 6). Relevant to these findings, bleomycin induced the formation of immature vascular networks consisting of tiny vessels, which was also attenuated by AdipoRon treatment (Fig. 6a). Viewed together, these results indicate that AdipoRon has beneficial effects on bleomycin-induced dermal microvascular damage by preventing EndoMT and promoting vessel stabilization.

### AdipoRon mitigates the intrinsic fibrotic phenotype of SSc fibroblasts

**Figure 7:**
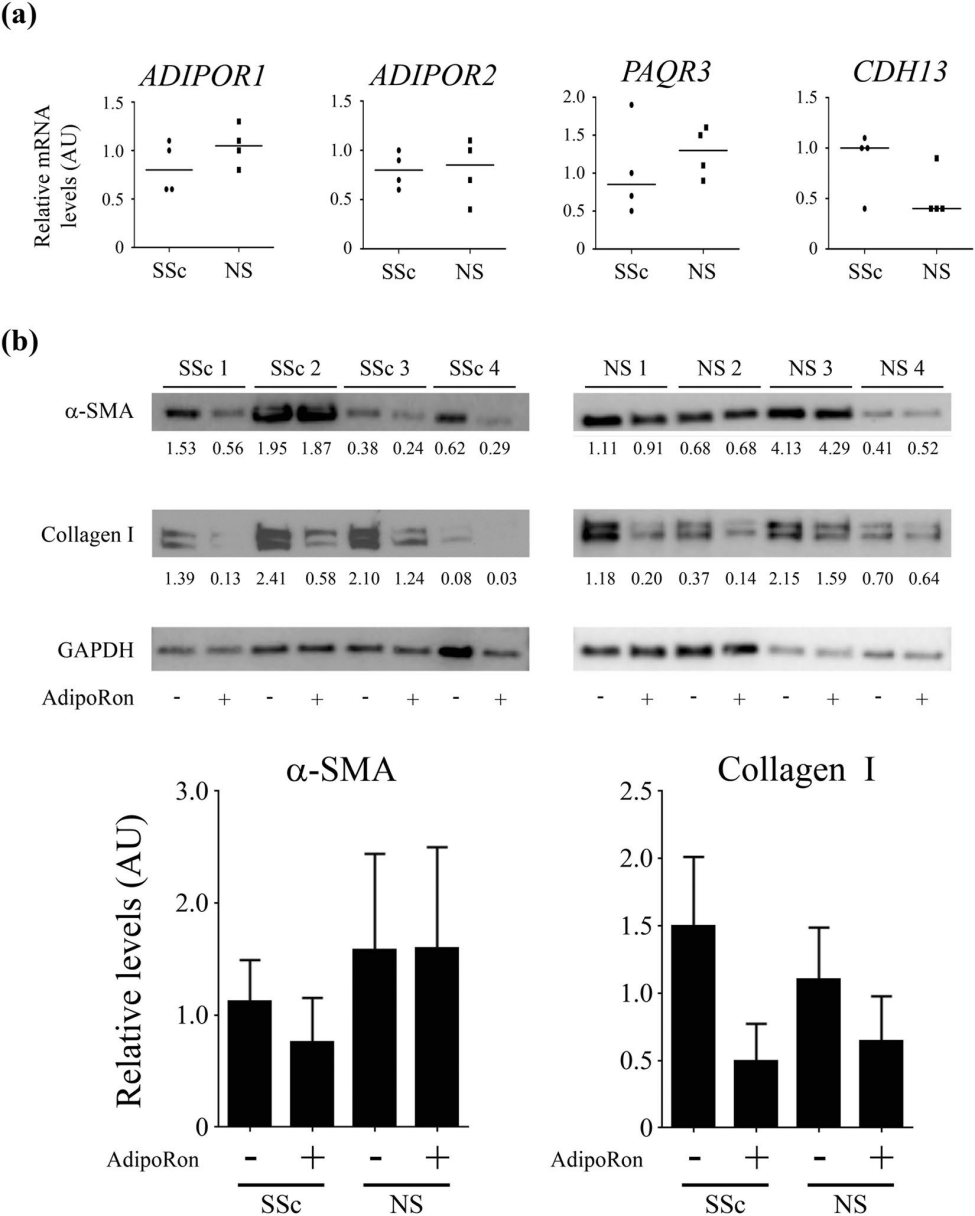
AdipoRon *ex vivo* reverses the fibrotic phenotype of SSc dermal fibroblasts. (**a**) mRNA levels of adiponectin receptors were determined by qRT-PCR in dermal fibroblasts isolated from SSc lesional skin and control normal skin (NS). Horizontal bars represent medians. (**b**) Confluent dermal fibroblasts isolated from SSc lesional skin (n = 4) and control normal skin (n = 4) were incubated with AdipoRon (20 μM) for 24 h. Whole cell lysates were subjected to immunoblotting. Representative blots of three independent experiments are shown. The values below each blot represent relative levels of target molecules normalized by loading controls with densitometry. Bars represent mean ± SD of indicated parameters.

All four known adiponectin receptors (AdipoR1, AdipoR2, AdipoR3, and T-cadherin) were found to be expressed in SSc fibroblasts at levels comparable to those in normal fibroblasts (Fig. 7a). Treatment with AdipoRon *ex vivo* resulted in variable suppression of fibrotic gene expression in SSc and normal fibroblasts, but the effects were considerably stronger in SSc fibroblasts (Fig. 7b), especially for α-SMA (the fold induction, 0.61 ± 0.13 versus 1.04 ± 0.09 [p < 0.05] for α-SMA and 0.32 ± 0.11 versus 0.55 ± 0.17 [p = 0.30] for type I collagen). In foreskin fibroblasts, treatment with TGF-β1 or ectopic expression of Egr-1 had no effect on expression of adiponectin receptors (Supplementary Table 3). These results indicate that AdipoRon exerts its anti-fibrotic effects on dermal fibroblasts irrespective of their activation status.

## Discussion

In view of the emerging importance of adiponectin in modulating a broad range of cellular activities implicated in SSc pathogenesis, we sought to investigate the impact of AdipoRon, the first orally-active AdipoR agonist, in a preclinical model of SSc. We found that AdipoRon treatment prevented fibrosis by attenuating fibroblast activation, adipocyte and endothelial cell transition to myofibroblasts, Th2/Th17-skewed immune polarization, and vascular activation. Importantly, the expression of AdipoR1 and AdipoR2 was increased by bleomycin treatment in multiple cell types in the skin, while adiponectin expression is generally suppressed in bleomycin-treated skin^6^. Therefore, activation of adiponectin receptor signaling might represent a plausible strategy to attenuate SSc-related disease processes, a notion supported by a series of experiments with AdipoRon.

Increased number of myofibroblasts within the lesional tissue is a histopathological hallmark of SSc shared with other fibrotic disorders^8^. Myofibroblasts originate from resident fibroblasts, as well as via transition of epithelial cells, endothelial cells, pericytes and adipocytic progenitor cells^6,40,45,46^. In bleomycin-treated mice, transition of endothelial cells and adipocytes to myofibroblasts were both attenuated by AdipoRon. Since myofibroblast conversion of adipocytes and/or endothelial cells has been implicated in tissue fibrosis of the skin, lung, heart, kidney, and pulmonary artery^40,45–51^, inhibition of myofibroblast accumulation is likely to represent a key mechanism underlying the potent anti-fibrotic effects of adiponectin.

Adiponectin modulation of the Th cytokine balance has been implicated in autoimmune and inflammatory conditions. For instance, in collagen-induced murine arthritis adiponectin augments articular bone destruction by enhancing Th17 response^52^, while in asthma it exacerbates respiratory inflammation via its anti-Th1 effect^53^. Taken together with the current finding that AdipoRon suppresses Th2/Th17 responses in bleomycin-treated mice, adiponectin receptor signaling seems to modulate the Th balance in a context-dependent manner. Importantly, the regulatory effect of adiponectin receptor signaling on Th2/Th17 responses is consistent with a datum regarding Th cell subsets and adiponectin levels in SSc. In early dcSSc pro-fibrotic Th2/Th17 responses are predominant^54,55^, while the Th balance shifts towards an anti-fibrotic Th1 response along with the resolution of skin sclerosis^56,57^. Serum adiponectin levels are decreased in early, but not in late-stage dcSSc and increased in patients treated with anti-fibrotic therapy^4,11,12^. Therefore, decreased adiponectin might contribute to SSc fibrosis via pro-fibrotic inflammation as well as fibroblast activation.

Vasculopathy in SSc encompasses vascular activation and structural abnormalities, both of which contribute to tissue fibrosis^58^. AdipoRon modulates both of these two aspects of SSc vasculopathy. A hallmark of SSc vascular activation is up-regulation of ICAM-1 in endothelial cells^40,59^, and ICAM-1 deficiency significantly attenuates bleomycin-induced dermal fibrosis by decreasing inflammatory Th2/Th17 cell accumulation^39^. Since AdipoRon reduced bleomycin-induced dermal fibrosis in parallel with decreased Th2/Th17 cell infiltration and endothelial ICAM-1 down-regulation, adiponectin receptor signaling likely inhibits a pro-fibrotic inflammatory response through vascular inactivation. With respect to structural abnormalities, capillary dilation and arteriolar stenosis are evident in SSc due to vascular destabilization and intimal fibro-proliferation, respectively^58^. Since AdipoRon attenuated bleomycin-induced vascular leakage and loss of vascular integrity, decreased adiponectin expression may underlie vascular destabilization in SSc. Adiponectin selectively accumulates in injured vessel walls by binding specifically to collagen types I, III, V and VIII of the vascular intima, and may contribute to vascular repair^60^. Considering the regulatory role of adiponectin in tissue fibrosis, decreased adiponectin expression may promote the fibro-proliferative changes of arterioles in SSc. Thus, adiponectin receptor agonists might exert a protective effect on SSc vasculopathy, possibly leading to the attenuation of tissue fibrosis.

AdipoRon is thought to bind exclusively to AdopoR1 and AdopoR2^30^, while adiponectin also activates AdipoR3^61^. Both AdipoR1 and AdipoR2 contain seven transmembrane domains but, in contrast to other G-protein-coupled receptors, they present an extracellular C and a cytosolic N terminus^62^. Recent resolution of the crystal structure of AdipoR1 and AdipoR2 is expected to lead to increasing insights into the regulation of adiponectin signaling^63^. Pharmacological approaches to augment adiponectin signaling have focused on boosting adiponectin levels or augmenting activity with receptor agonists. Adiponectin levels can be increased by treatment with thiazolidinediones, as well as dietary compounds such as curcumin, gingerol, capsaicin, and omega-3 fatty acids^64,65^. Naturally occurring adiponectin receptor agonists include osmontin, a plant-derived protein resembling the β barrel of adiponectin domain I^66^. Screening of small molecular plant libraries have also identified matairesinol, arctiin, (-)-arctigenin and gramine as AdipoR1 agonists, while parthenolide, taxifoliol, deoxyschizandrin, and syringin are AdipoR2 agonists^67^. Undesirable potential effects of adiponectin such as reduced bone density, left ventricular hypertrophy, infertility and growth of established tumors due to enhanced angiogenesis^68^, are concerns for all AdipoR agonists.

The present studies are significant since they represent the first to demonstration for the pleiotropic anti-fibrotic effects of AdipoRon *in vitro* and *in vivo*. In view of its potent beneficial impact on all three cardinal pathomechanisms underlying the clinical manifestations of SSc, adiponectin receptor agonists represent promising novel therapeutic approaches for SSc.

## Materials and Methods

### Ethics Statement

The study was performed according to the Declaration of Helsinki and approved by Institutional Review Board for Human Studies at Northwestern University and by the ethical committee of The University of Tokyo Graduate School of Medicine. Written informed consent was obtained from SSc patients, healthy donors, or their parents/legal guardians.

### Animal experiments

Bleomycin (Nippon Kayaku, Tokyo, Japan) was dissolved in PBS at the concentration of 1 mg/ml and sterilized by filtration, and 200 μg was injected subcutaneously into the shaved backs of C57BL/6 wild-type female mice (Japan SLC Inc., Tokyo, Japan). AdipoRon (Arking Pharma Scientific Inc., ON, Canada) was suspended in aqueous solution of 0.5% carboxy methyl cellulose. AdipoRon (50 mg/kg) or vehicle was administered orally together with s.c. bleomycin. Mice were harvested at indicated time points. All animal protocols were approved by the Animal Care and Use Committee of University of Tokyo (approval ID: P14-064), and all methods were performed in accordance with the relevant guidelines and regulations.

### Histological assessment and immunostaining

All skin sections were taken from the paramidline, lower back region. Sections were stained with hematoxylin and eosin (H&E) and toluidine blue. We examined dermal thickness, which was defined as the thickness of skin from the top of the granular layer to the junction between the dermis and subcutaneous fat. Immunohistochemistry and immunofluorescence were performed with antibodies against α-SMA (Sigma-Aldrich, St. Louis, MO, USA), ICAM-1 (BD Pharmingen, San Diego, CA, USA), VE-cadherin (Santa Cruz Biotechnology, Santa Cruz, CA, USA), FSP-1 (Abcam, Cambridge, MA, USA), perilipin (Santa Cruz Biotechnology), AdipoR1 (Abcam), AdipoR2 (Santa Cruz Biotechnology) and PDGFR-β (R&D Systems, Minneapolis, MN, USA) as described previously^45^. All sections were examined independently by two investigators in a blinded manner. To quantify signal intensity (ICAM-1 and α-SMA), color images were converted to grayscale, and brightness of vessels was measured in 5 different randomly selected vessels per specimen using ImageJ.

### Determination of hydroxyproline content in skin

Following instructions of the QuickZyme Total Collagen Assay kit (QuickZyme Biosciences, Leiden, Netherlands), 6-mm punch biopsy skin samples were hydrolyzed with 6 N HCl and collagen content was quantified.

### Quantitative real-time reverse transcription PCR

Gene expression levels were determined by quantitative real-time reverse transcription PCR (qRT-PCR), as described previously^69^. mRNA levels of target genes were normalized to those of the *GAPDH* gene or the *Gapdh* gene. The sequences of primers were summarized in Supplementary Table 4.

### Flow cytometric analysis

WT mice were treated with bleomycin for 7 days in the presence or absence of AdipoRon, as described above. The next day, lymphocytes from lymph nodes draining the lesional skin were obtained. For intracellular cytokine staining experiments, cells were stimulated with 10 ng/ml of phorbol myristate acetate and 1 μg/ml of ionomycin (Sigma-Aldrich), in the presence of 1 μg/ml of brefeldin A (GolgiStop; BD Pharmingen) for 4 h. Staining was performed according to the protocol of the anti-mouse/rat Foxp3 staining set (eBioscience, San Diego, CA), using anti-CD4 (RM4-5; BioLegend, San Diego, CA), anti-CD25 (PC01; BioLegend), anti-IL-4 (11B11; BioLegend), anti-IL-17A (TC11.18H10; BioLegend), anti-IFN-γ (XMG1.2; BioLegend), and anti-Foxp3 (FJK-16s; eBioscience) antibodies. Cells were analyzed on a FACSVerse flow cytometer (BD Biosciences, San Jose, CA).

### Cell cultures

Primary fibroblasts (passage 3–6) established with explantation from neonatal foreskin fibroblasts or skin biopsies from forearm of healthy adults and SSc patients were used. Cells were cultured in DMEM supplemented with 1% glutamine, 50 µg/ml penicillin/streptomycin, 10% fetal bovine serum, 1% vitamin solutions and 2 mM L-glutamine in humidified atmosphere of 5% CO_2_ at 37 °C until confluence. All cell culture reagents were from Lonza (Basel, Switzerland). For experiments, fibroblasts were placed in serum-free media containing 0.1% bovine serum albumin (BSA) for 24 h prior to addition of AdipoRon (Sigma-Aldrich) dissolved in DMSO. Cultures were preincubated with AdipoRon and or compound C (Sigma-Aldrich) for 60 min prior to TGF-β1 (PeproTech, Rocky Hill, NJ). Toxicity was determined using lactate dehydrogenase assay according to manufacturer instructions (Biovisison, Milpitas, CA, USA).

### Human dermal microvascular endothelial cells

HDMECs were purchased from Lonza Ltd. (Basel, Switzerland). Confluent cultures of HDMECs were treated with AdipoRon at the indicated concentrations for 60 min prior to 0.5 ng/ml of TNF-α. Total RNA was isolated 24 h later from cell lysates as described above.

### Immunoblotting

Fibroblasts were harvested, whole-cell lysates were prepared with RIPA buffer and proteinase/phosphatase inhibitor cocktail and equal amounts of proteins (20–50 µg/lane) were subjected to immunoblotting as described previously^5^. Antibodies specific for human type I collagen (Southern Biotechnology, Birmingham, AL, USA), α-SMA (Sigma -Aldrich), tubulin (Sigma-Aldrich), GAPDH (Zymed, San Francisco, CA, USA), fibronectin (Santa Cruz Biotechnology), p-AMPK, AMPK (Cell Signaling, Danvers, MA. USA), AdipoR1 and AdipoR2 were used as primary antibodies with overnight incubation. After washing membranes were incubated with appropriate secondary antibodies and subjected to enhanced chemiluminescence detection using ECL Reagent (Pierce, Rockford, IL, USA).

### Scratch assays

Fibroblast function was observed using scratch assay. Confluent foreskin fibroblast monolayers were mechanically wounded using p1000 pipette tips. Following 24-hour incubation of the cultures wound gap widths were determined at six randomly selected sites/high power field.

### Immunofluorescence confocal microscopy

Fibroblasts seeded on 8-well Lab-Tek II chamber glass slides (Nalgene Nunc International, Naperville, IL, USA) were incubated in serum-free DMEM with 0.1% BSA for 24 h. Fresh media with AdipoRon were added for 24 h. Cells were fixed, permeabilized, and incubated with primary antibodies to α-SMA (Sigma-Aldrich) and type I collagen, both at 1:500 followed by Alexa-fluor-labelled secondary antibodies (Invitrogen, Carlsbad, CA, USA). Nuclei were identified using 4,6-diamidino-2-phenylindone (DAPI) in ProLong Gold antifade reagent (Invitrogen). Immunofluorescence was evaluated under a Zeiss UV Meta 510 confocal microscope (Carl Zeiss Inc, Jena, Germany).

### Statistical analysis

Statistical analysis was done with one-way ANOVA followed by Turkey *post hoc* test for multiple comparisons and with Mann-Whitney U-test to compare the distributions of two unmatched groups. Statistical significance was defined as a P value of < 0.05.

## Electronic supplementary material

Supplementary Table
