# Supplementary material for: An orally-active adiponectin receptor agonist mitigates cutaneous fibrosis, inflammation and microvascular pathology in a murine model of systemic sclerosis
Source: Sci Rep. 2018 Aug 7;8:11843. doi: 10.1038/s41598-018-29901-w (PMC6081386; doi:10.1038/s41598-018-29901-w)
Supplement: Supplementary file 1 — Supplementary Table [file 41598_2018_29901_MOESM1_ESM.pdf]

**An orally-active adiponectin receptor agonist mitigates cutaneous fibrosis, inflammation and microvascular pathology in a murine model of systemic sclerosis**

Takashi Yamashita[1]*, Katja Lakota[2,3]*, Takashi Taniguchi[1], Ayumi Yoshizaki[1], Shinichi Sato[1], Wen Hong[3], Xingchun Zhou[3], Snezna Sodin-Semrl[2], Feng Fang[3], Yoshihide Asano[1], John Varga[3].

[1]Department of Dermatology, University of Tokyo Graduate School of Medicine, Tokyo, Japan

[2]Department of Rheumatology, University Medical Centre Ljubljana, Slovenia

[3]Northwestern Scleroderma Program, Feinberg School of Medicine, Chicago, IL

* T.Y. and K.L. contributed equally to this work.

**Supplementary Table 1. Cell-specific expression of CTGF in lesional skin.**

| Treatment | Fibroblasts | Immune cells | Endothelial cells | Adipocytes |
|---|---|---|---|---|
| PBS + VE, 1 | - | - | - | + |
| PBS + VE, 2 | + | + | + | + |
| PBS + VE, 3 | - | + | - | - |
| PBS + VE, 4 | + | - | - | - |
| PBS + VE, 5 | + | - | - | - |
| PBS + ARon, 1 | - | + | - | - |
| PBS + ARon, 2 | - | - | - | - |
| PBS + ARon, 3 | + | - | + | - |
| PBS + ARon, 4 | + | - | - | + |
| PBS + ARon, 5 | + | - | - | - |
| BLM + VE, 1 | +++ | ++ | ++ | + |
| BLM + VE, 2 | +++ | ++ | - | ++ |
| BLM + VE, 3 | ++ | + | + | - |
| BLM + VE, 4 | +++ | +++ | + | + |
| BLM + VE, 5 | +++ | ++ | +++ | + |
| BLM + ARon, 1 | + | + | ++ | + |
| BLM + ARon, 2 | + | - | - | - |
| BLM + ARon, 3 | + | - | - | - |
| BLM + ARon, 4 | + | + | + | + |
| BLM + ARon, 5 | + | + | - | + |

Immunostaining of murine skin sections with sequentially titrated antibodies against CTGF. Signal intensity in sections stained with 100-fold diluted anti-CTGF antibody was

quantified in which the difference of signal intensity was clearly detected among the four groups. Following grading system was used: -, no staining; +, slight staining; ++, moderate staining; +++, strong staining. BLM; bleomycin, ARon, AdipoRon; VE, vehicle.

**Supplementary Table 2. Expression of AdipoR1 and AdipoR2 in lesional skin.**

**AdipoR1**

| Treatment | Fibroblasts | Immune cells | Endothelial cells | Adipocytes |
|---|---|---|---|---|
| PBS + VE, 1 | + | - | - | - |
| PBS + VE, 2 | + | - | - | - |
| PBS + VE, 3 | - | - | - | - |
| PBS + VE, 4 | - | - | - | - |
| PBS + VE, 5 | - | - | - | - |
| PBS + ARon, 1 | + | - | - | - |
| PBS + ARon, 2 | - | - | - | - |
| PBS + ARon, 3 | - | - | - | - |
| PBS + ARon, 4 | - | - | - | - |
| PBS + ARon, 5 | - | - | - | - |
| BLM + VE, 1 | +++ | + | + | N.D. |
| BLM + VE, 2 | +++ | + | +++ | + |
| BLM + VE, 3 | +++ | + | + | N.D. |
| BLM + VE, 4 | +++ | +++ | ++ | + |
| BLM + VE ,5 | +++ | +++ | + | N.D. |
| BLM + ARon, 1 | +++ | +++ | +++ | + |
| BLM + ARon, 2 | ++ | + | + | N.D. |
| BLM + ARon, 3 | + | +++ | + | + |
| BLM + ARon, 4 | +++ | + | + | ++ |
| BLM + ARon, 5 | + | + | - | N.D. |

**AdipoR2**

| Treatment | Fibroblasts | Immune cells | Endothelial cells | Adipocytes |
|---|---|---|---|---|
| PBS + VE, 1 | + | - | - | - |
| PBS + VE, 2 | + | - | + | + |
| PBS + VE, 3 | - | - | - | - |
| PBS + VE, 4 | + | - | - | + |
| PBS + VE, 5 | - | - | - | - |
| PBS + ARon, 1 | + | - | + | + |
| PBS + ARon, 2 | - | - | - | - |
| PBS + ARon, 3 | + | - | - | - |
| PBS + ARon, 4 | - | - | - | - |
| PBS + ARon, 5 | - | - | - | - |
| BLM + VE, 1 | + | ++ | + | N.D. |
| BLM + VE, 2 | + | + | + | + |
| BLM + VE, 3 | + | + | + | + |
| BLM + VE, 4 | + | ++ | + | + |
| BLM + VE, 5 | + | ++ | + | N.D. |
| BLM + ARon, 1 | ++ | +++ | ++ | + |
| BLM + ARon, 2 | + | - | + | N.D. |
| BLM + ARon, 3 | + | + | + | + |
| BLM + ARon, 4 | + | + | + | + |
| BLM + ARon, 5 | + | + | + | + |

Immunostaining of murine skin sections with sequentially titrated antibodies against AdipoR1 or AdipoR2. Signal intensity in sections stained with 250-fold diluted primary antibodies was quantified in which the difference of signal intensity was clearly detected among the four groups. Following grading system was used: -, no staining; +, slight

staining; ++, moderate staining; +++, strong staining. Adipose tissue was not detected in some skin sections of bleomycin-treated mice because it was replaced with fibrotic tissues (shown as N.D. [not determined]). BLM; bleomycin, ARon, AdipoRon; VE, vehicle.

**Supplementary Table 3. Adiponectin receptor expression is not inducible by profibrotic stimuli.**

| | Control | | Egr | | TGF | |
|---|---|---|---|---|---|---|
| | 24h | 48h | 24h | 48h | 24h | 48h |
| AdipoR1 | 8.6 | 8.6 | 8.6 | 8.9 | 8.5 | 8.5 |
| | 8.6 | 8.6 | 8.8 | 8.8 | 8.6 | 8.6 |
| AdipoR2 | 9.9 | 10.6 | 10 | 10.6 | 9.8 | 10.5 |
| | 9.8 | 10.6 | 10 | 10.7 | 9.8 | 10.5 |
| PAQR3 | 8.1 | 8.1 | 8.3 | 8.4 | 7.9 | 8.3 |
| | 7.9 | 8.1 | 8.3 | 8.6 | 8.2 | 8 |
| T cadherin | 7.4 | 8.1 | 7.3 | 7.2 | 7.5 | 7.9 |
| | 7.5 | 8.2 | 7.4 | 7.2 | 7.5 | 7.9 |
| AdipoQ | 6.5 | 6.4 | 6.5 | 6.5 | 6.5 | 6.4 |
| | 6.5 | 6.4 | 6.4 | 6.5 | 6.5 | 6.4 |
| Col 1 | 13.9 | 14.1 | 14.4 | 14.5 | 13.9 | 14.1 |
| | 13.9 | 14.1 | 14.4 | 14.4 | 13.8 | 14 |

two samples per group

Human foreskin fibroblasts were infected with Ad-EGFP, or incubated with TGF-β in parallel. Total RNA was harvested after 48 h and subjected to microarray analysis using Illumina chips. Numbers in tables represent signal intensity with two independent samples for each gene. Col1 data are shown as representative of highly inducible signal, while adipoQ data are shown as case of signal for non-expressing gene. Data from GEO:GSE9285 (Bhattacharyya S, et al. PLoS One. 2011;6:e23082.)

**Supplementary Table 4. Sequences of primers used for qRT-PCR.**

Mouse primer

| | | |
|---|---|---|
| *Col1a1* | Forward: | 5'-GCCAAGAAGACATCCCTGAAG-3' |
| | Reverse: | 5'-TGTGGCAGATACAGATCAAGC-3' |
| *Col1a2* | Forward: | 5'-GGAGGGAACGGTCCACGAT-3' |
| | Reverse: | 5'-GAGTCCGCGTATCCACAA-3' |
| *Mmp13* | Forward: | 5'-TGATGGCACTGCTGACATCAT-3' |
| | Reverse: | 5'-TGTAGCCTTTGGAACTGCTT-3' |
| *Ctgf* | Forward: | 5'-GTGCCAGAACGCACACTG-3' |
| | Reverse: | 5'-CCCCGGTTACACTCCAAA-3' |
| *Tgfb1* | Forward: | 5'-GCAACATGTGGAACTCTACCAGAA-3' |
| | Reverse: | 5'-GACGTCAAAAGACAGCCACTCA-3' |
| *Tnfa* | Forward: | 5'-ACCCTCACACTCAGATCATCTTC-3' |
| | Reverse: | 5'-TGGTGGTTTGCTACGACGT-3' |
| *Il1b* | Forward: | 5'-TTGACGGACCCCAAAAGAT-3' |
| | Reverse: | 5'-GAAGCTGGATGCTCTCATCTG-3' |
| *Ccl2* | Forward: | 5'-CATCCACGTGTTGGCTCA-3' |
| | Reverse: | 5'-GATCATCTTGCTGGTGAATGAGT-3' |
| *Ifng* | Forward: | 5'-TCAAGTGGCATAGATGTGGAAGAA-3' |
| | Reverse: | 5'-TGGCTCTGCAGGATTTTCATG-3' |
| *Il4* | Forward: | 5'-ACGGAGATGGATGTGCCAAACGTC-3' |
| | Reverse: | 5'-CGAGTAATCCATTTGCATGATGC-3' |
| *Il6* | Forward: | 5'-GATGGATGCTACCAAACTGGAT-3' |
| | Reverse: | 5'-CCAGGTAGCTATGGTACTCCAGA-3' |
| *Il10* | Forward: | 5'-TTTGAATTCCCTGGGTGAGAA-3' |
| | Reverse: | 5'-ACAGGGGAGAAATCGATGACA-3' |
| *Il13* | Forward: | 5'-GCAACATCACACAAGACCAGA-3' |
| | Reverse: | 5'-GTCAGGGAATCCAGGGCTAC-3' |
| *Il17a* | Forward: | 5'-CTCCAGAAGGCCCTCAGACTAC-3' |
| | Reverse: | 5'-AGCTTTCCCTCCGCATTGACACAG-3' |
| *Arg1* | Forward: | 5'-CAGAAGAATGGAAGAGTCAG-3' |
| | Reverse: | 5'-CAGATATGCAGGGAGTCACC-3' |
| *Fizz1* | Forward: | 5'-TCCCAGTGAATACTGATGAGA-3' |
| | Reverse: | 5'-CCACTCTGGATCTCCCAAGA-3' |

| | | |
|---|---|---|
| *Ym1* | Forward: | 5'-GGGCATACCTTTATCCTGAG-3' |
| | Reverse: | 5'-CCACTGAAGTCATCCATGTC-3' |
| *Icam1* | Forward: | 5'-GACGCAGAGGACCTTAACAG-3' |
| | Reverse: | 5'-GACGCCGCTCAGAAGAAC-3' |
| *Glycam1* | Forward: | 5'-GACGCAGAGGACCTTAACAG-3' |
| | Reverse: | 5'-GACGCCGCTCAGAAGAAC-3' |
| *Selp* | Forward: | 5'-TCCAGGAAGCTCTGACGTACTTG-3' |
| | Reverse: | 5'-GCAGCGTTAGTGAAGACTCCGTAT-3' |
| *Sele* | Forward: | 5'-TGAACTGAAGGGATCAAGAAGACT-3' |
| | Reverse: | 5'-GCCGAGGGACATCATCACAT-3' |
| *Snai1* | Forward: | 5'-CAACTATAGCGAGCTGCAGGA-3' |
| | Reverse: | 5'-ACTTGGGGTACCAGGAGAGAGT-3' |
| *Gapdh* | Forward: | 5'-CGTGTTCCTACCCCCAATGT-3' |
| | Reverse: | 5'-TGTCATCATACTTGGCAGGTTTCT-3' |

Human primer

| | | |
|---|---|---|
| *ASMA* | Forward: | 5'-CAGGGCTGTTTTCCCATCCAT-3' |
| | Reverse: | 5'-GCCATGTTCTATCGGGTACTTC-3' |
| *COL1A1* | Forward: | 5'-TGGTGTGCAAGGTCCC-3' |
| | Reverse: | 5'-CATTCCCTGAAGGCCAG-3' |
| *MMP1* | Forward: | 5'-GCACAAATCCCTTCTACCCG-3' |
| | Reverse: | 5'-TGAACAGCCCAGTACTTATTCC-3' |
| *ICAM1* | Forward: | 5'-TAGAGACCCCGTTGCCTAAA-3' |
| | Reverse: | 5'-TCATACACCTTCCGGTTGTTC-3' |
| *SNAI1* | Forward: | 5'-ACCCCAATCGGAAGCCTAACT-3' |
| | Reverse: | 5'-GGTCGTAGGGCTGCTGGAA-3' |
| *ADIPOR1* | Forward: | 5'-AAACTGGCAACATCTGGACC-3' |
| | Reverse: | 5'-GCTGTGGGGAGCAGTAGAAG-3' |
| *ADIPOR2* | Forward: | 5'-ACAGGCAACATTTGGACACA-3' |
| | Reverse: | 5'-CCAAGGAACAAAACTTCCCA-3' |
| *GAPDH* | Forward: | 5'-CATGAGAAGTATGACAACAGCCT-3' |
| | Reverse: | 5'-AGTCCTTCCACGATACCAAAGT-3' |

## Supplementary Figure 1

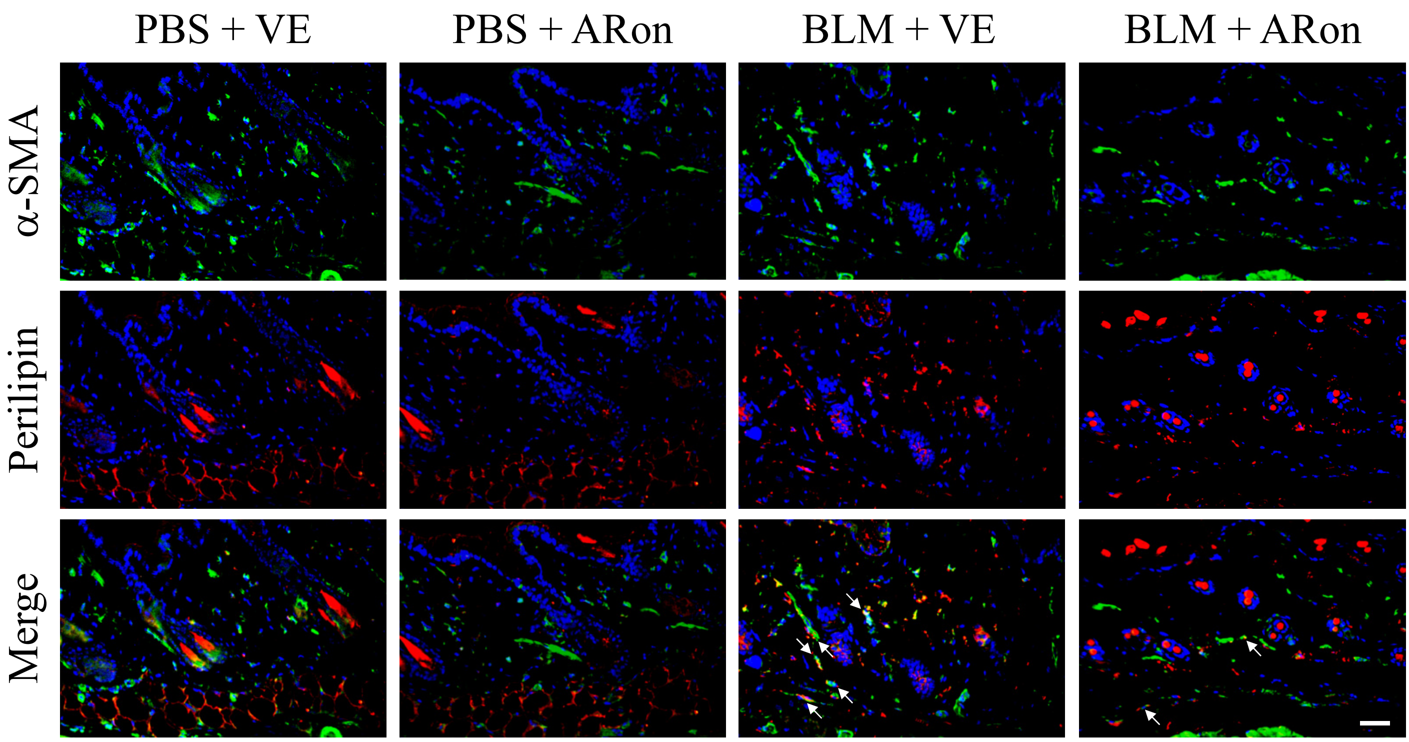


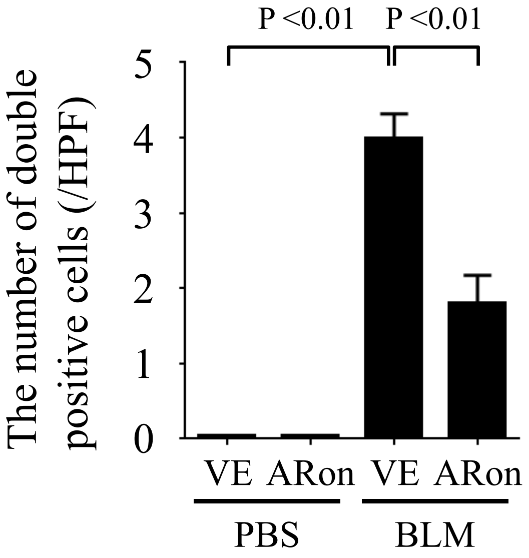

## Supplementary Figure 2

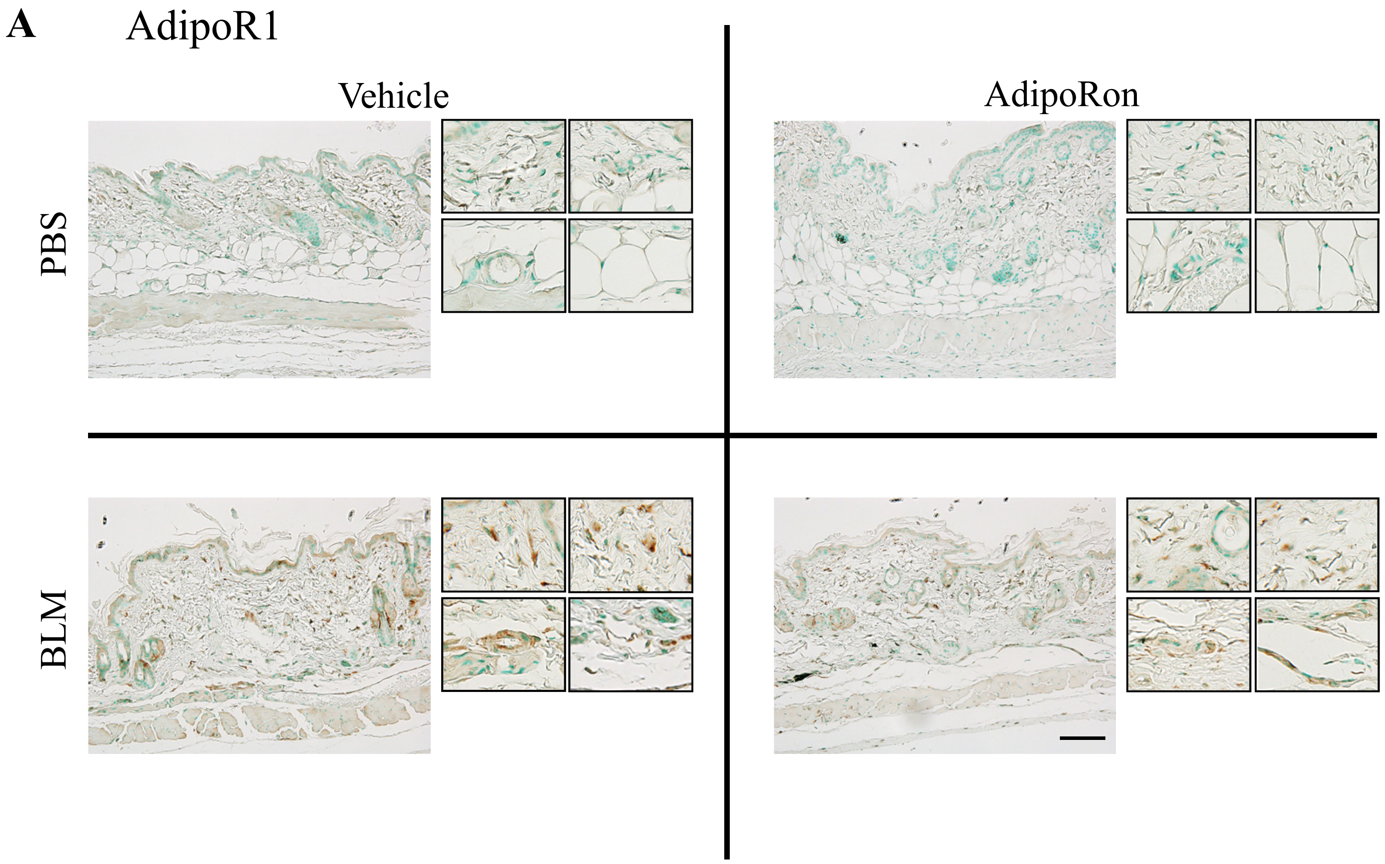


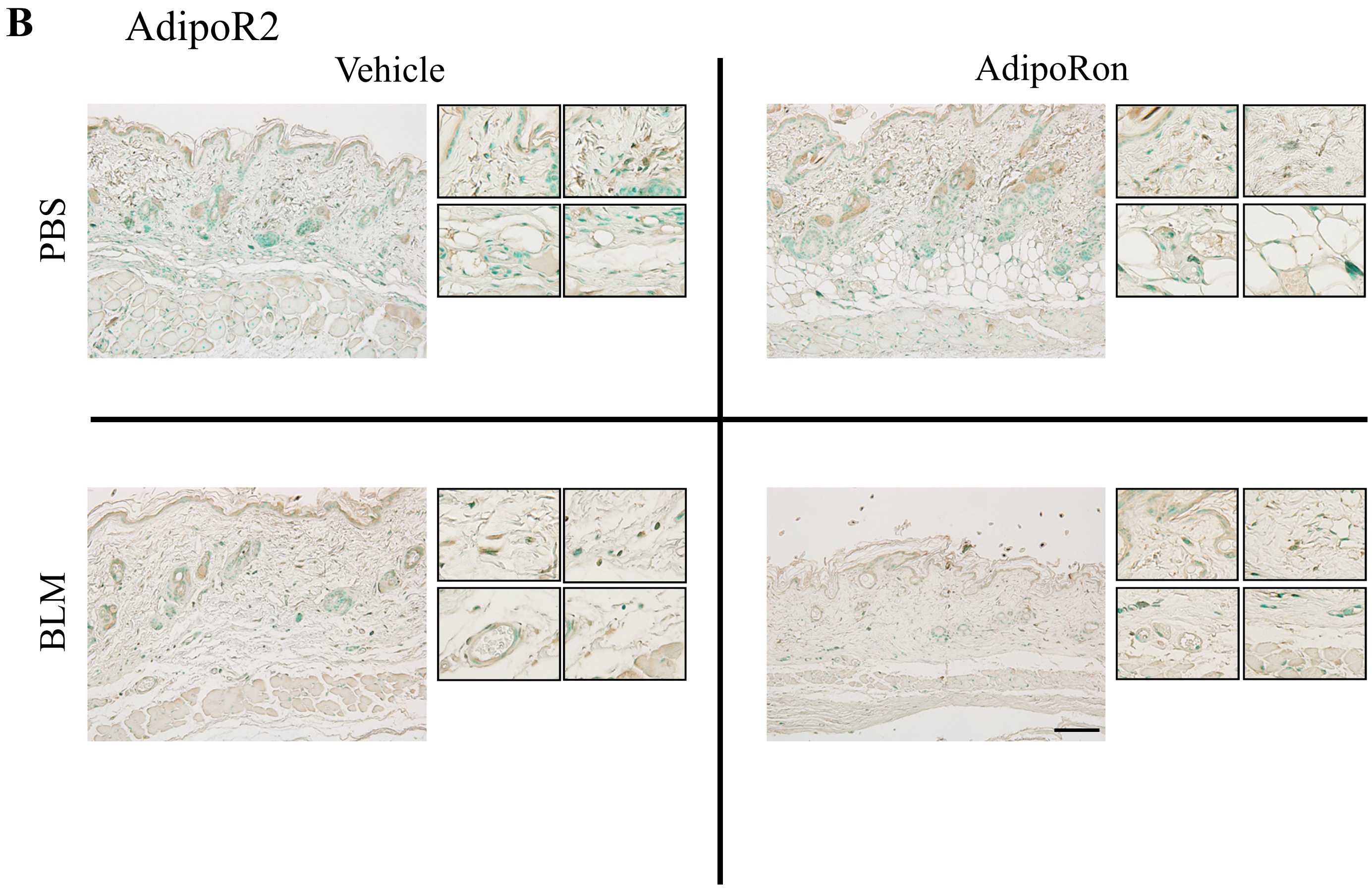

**Supplementary Figure 3**

**A**

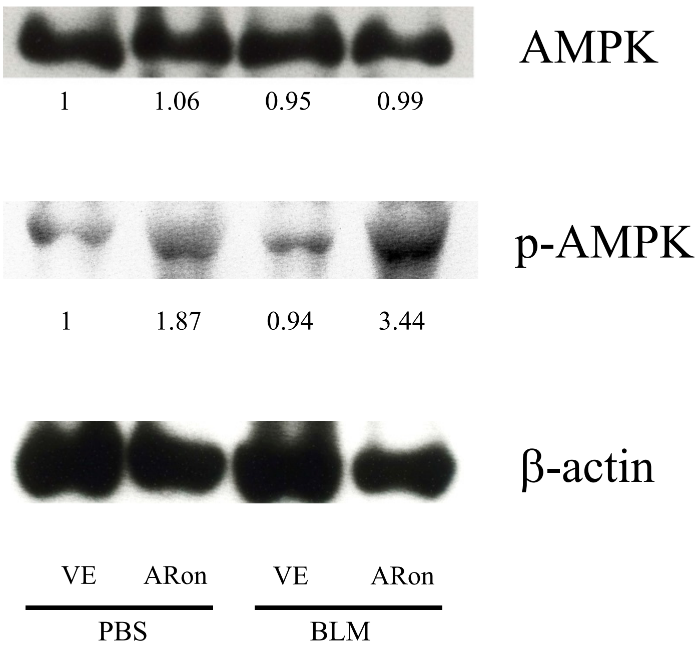


**B**

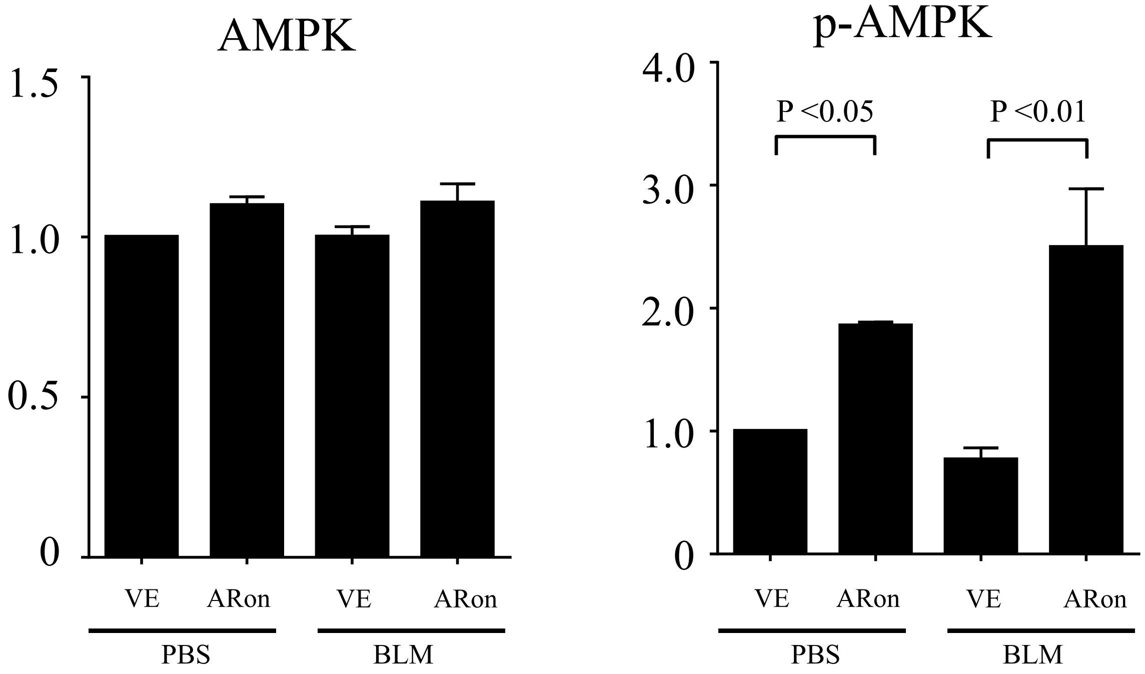

**Supplementary Figure 4**

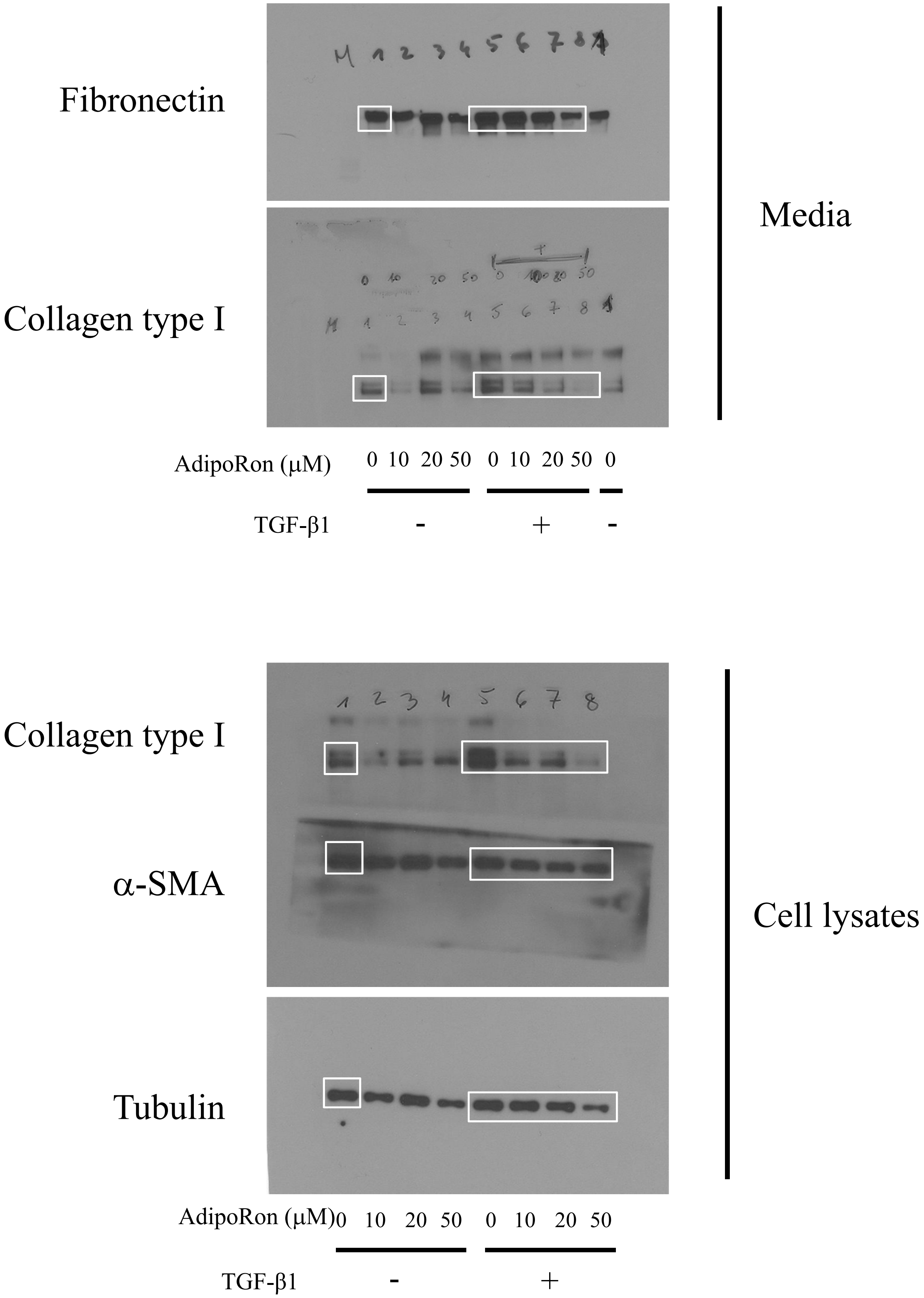

## Supplementary Figure 5

**A**

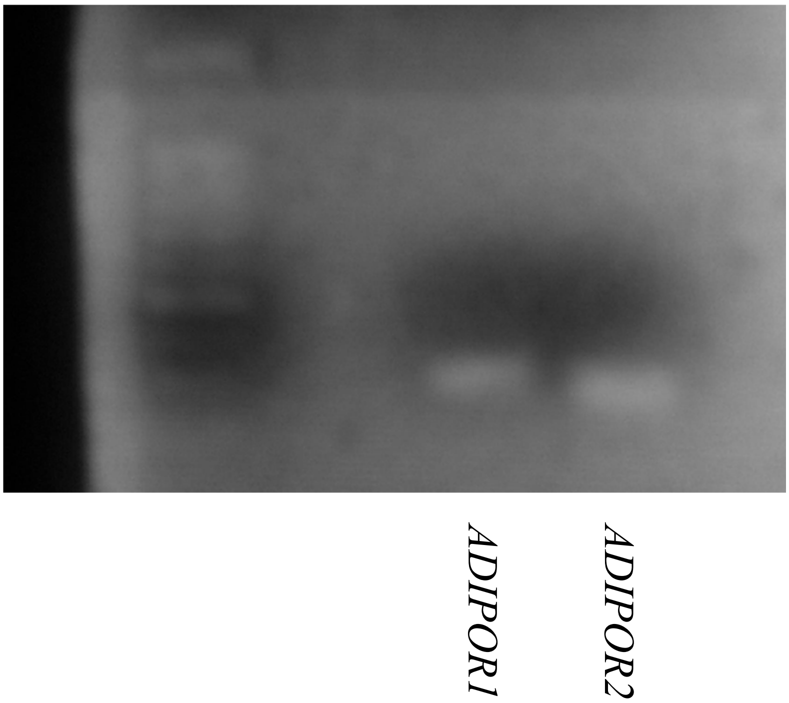


**B**

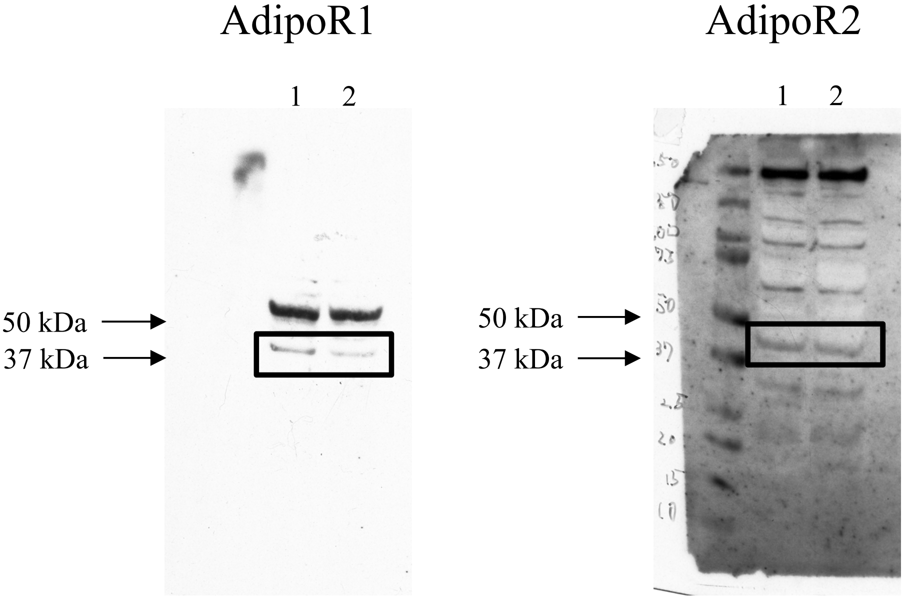

**Supplementary Figure 6**

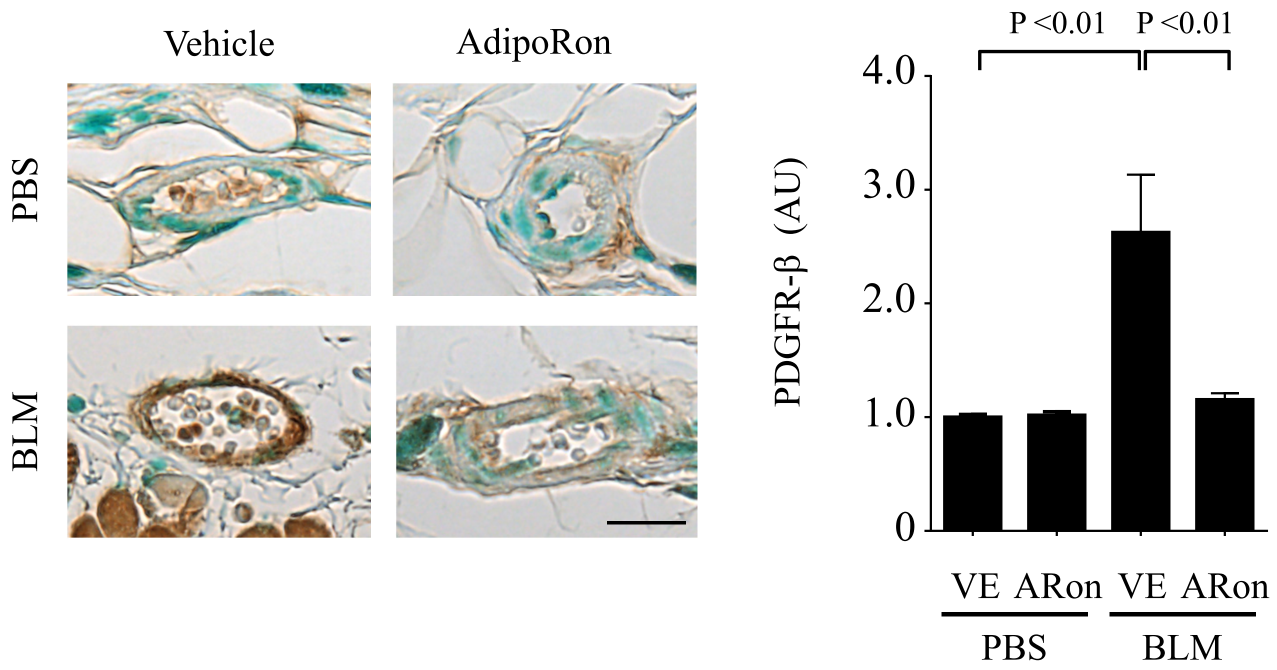

**Figure Legends**

**Supplementary figures.**

**Supplementary Figure 1. AdipoRon treatment attenuates transdifferentiation of intradermal adipocytes to myofibroblasts in the lesional skin.**

Mice were treated with bleomycin (BLM) or PBS for 4 weeks alone or combined with AdipoRon. Immunofluorescence staining of α-SMA (green) and perilipin (red); α-SMA/perilipin double positive cells indicated with arrows. Representative images. The number of double-positive cells per high power field is shown (n=5). Scale bar, 20 μm. HPF, high power field; ARon, AdipoRon; VE, vehicle.

**Supplementary Figure 2. BLM induces AdipoR1 and AdipoR2 in multiple cell types in the lesional skin.**

Mice were treated with bleomycin (BLM) or PBS for 4 weeks in the presence or absence of AdipoRon. Skin sections were immunostained with anti-AdipoR1 (A) or anti-AdipoR2 antibody (B). Representative results are shown (n = 5). Fibroblasts are shown in left upper small panels. Immune cells are shown in right upper small panels. Endothelial cells are shown in left lower panels. Adipocytes are shown in right lower panels. Scale bars are 50 μm.

**Supplementary Figure 3. AdipoRon treatment induces AMPK phosphorylation in the skeletal muscle.**

Mice were treated with bleomycin (BLM) or PBS for 4 weeks alone or combined with AdipoRon. **A.** Quadriceps muscle were harvested, immersed in a RIPA buffer with proteinase/phosphatase inhibitor cocktail, finely crushed, and cell lysates with equal amounts of proteins (20 - 50 μg/lane) were subjected to immunoblotting (n = 3). Representative blots. The values below each blot represent the relative levels of target

molecules normalized to loading controls. **B.** Results of three independent experiments. ARon, AdipoRon; VE, vehicle.

**Supplementary Figure 4. Full-length blots of Figure 2D.**

Cropped areas used for Figure 2D are shown with white squares in full-length blots.

**Supplementary Figure 5. HDMECs express AdipoR1 and AdipoR2.**

**A.** Expression of *ADIPOR1* and *ADIPOR2* mRNA in HDMECs confirmed by PCR. Amplified products visualized by gel electrophoresis. **B.** Whole cell lysates prepared from HDMECs were subjected to immunoblotting. Bands corresponding to AdipoR1 and AdipoR2 shown as squares.

**Supplementary Figure 6. Expression of PDGFR-β in dermal small vessel pericytes.**

Mice were treated with bleomycin (BLM) or PBS for 4 weeks alone or combined with AdipoRon, and lesional skin was harvested. Immunostaining for PDGFR-β (n = 5 mice/group). Signal intensities analyzed by ImageJ. Scale bar, 20 μm. AU, arbitrary unit; ARon, AdipoRon; VE, vehicle.
